# Preparation and Characterization of Diamond-like Carbon Coatings for Biomedical Applications—A Review

**DOI:** 10.3390/ma16093420

**Published:** 2023-04-27

**Authors:** Klaudia Malisz, Beata Świeczko-Żurek, Alina Sionkowska

**Affiliations:** 1 Department of Biomaterials Technology, Faculty of Mechanical Engineering and Ship Technology, Gdansk University of Technology, Gabriela Narutowicza 11/12, 80-229 Gdansk, Poland; beata.swieczko-zurek@pg.edu.pl; 2 Faculty of Chemistry, Nicolaus Copernicus University in Toruń, Gagarina 7, 87-100 Torun, Poland

**Keywords:** diamond-like carbon, bioactive coating, implants, deposition techniques

## Abstract

Diamond-like carbon (DLC) films are generally used in biomedical applications, mainly because of their tribological and chemical properties that prevent the release of substrate ions, extend the life cycle of the material, and promote cell growth. The unique properties of the coating depend on the ratio of the sp^3^/sp^2^ phases, where the sp^2^ phase provides coatings with a low coefficient of friction and good electrical conductivity, while the share of the sp^3^ phase determines the chemical inertness, high hardness, and resistance to tribological wear. DLC coatings are characterized by high hardness, low coefficient of friction, high corrosion resistance, and biocompatibility. These properties make them attractive as potential wear-resistant coatings in many compelling applications, including optical, mechanical, microelectronic, and biomedical applications. Another great advantage of DLC coatings is that they can be deposited at low temperatures on a variety of substrates and can thus be used to coat heat-sensitive materials, such as polymers. Coating deposition techniques are constantly being improved; techniques based on vacuum environment reactions are mainly used, such as physical vapor deposition (PVD) and chemical vapor deposition (CVD). This review summarizes the current knowledge and research regarding diamond-like carbon coatings.

## 1. Introduction

Diamond-like carbon films are often used in biomedical applications, generally because of their mechanical and chemical properties. DLC films reduce ion release from the protected material, which prevents possible allergies and inflammatory reactions, as well as increases the material life cycle and supports cellular growth. Additionally, carbon layers have great resistance to bacterial colonization. Moreover, this type of coating is characterized by high hardness, a low friction coefficient, chemical inertness, good corrosion resistance, infrared transparency, a high refractive index, and excellent smoothness. However, several disadvantages, such as low thermal stability and high internal stress causing deficient adhesion, limit the usefulness of DLC coatings [1,2,3,4,5,6,7,8].

It is well known that diamond-like carbon films contain a mixture of sp^3^, sp^2^, and sp^1^-bonds of coordinated carbon disordered atoms. However, the properties of these coatings depend on the sp^3^ and sp^2^ bonding hybridization of the carbon atoms and the relative concentrations of these bonds [7,9,10]. Different deposition techniques have been proposed that give rise to a large selection of amorphous carbons with varying sp3 and hydrogen contents. The fraction of sp^3^ bonds and the hydrogen content significantly contribute to the structure and, consequently, the final properties of DLC films. Studies have demonstrated that the structure (sp^2^/sp^3^) has an important role in controlling the biological response of DLC and can improve the use of DLC films. DLC with a tendency toward diamond structure (more sp^3^ content) has higher biocompatibility than DLC with a tendency toward graphite structure (more sp^2^ content) [2,11,12,13]. This is due to the absence of repulsive forces. The ratio of the sp^3^/sp^2^ phases determines the classification of carbon coatings. The sp^2^ phase provides coatings with a low coefficient of friction and good electrical conductivity, and the sp^3^ phase provides chemical inertness, high hardness, and resistance to tribological wear [14,15].

Ag, F, P, Si, and Ti are a few chemical components that could be doped into DLC coatings, but silver is more commonly used because of its antibacterial properties, which are crucial for biomedical applications. Furthermore, DLC films doped with silver and gold nanoparticles are attractive because of the surface plasmon resonance effect. It allows them to be applied in nanoscale sensors and devices used as ultrasensitive chemical and biological sensors [1,3,5]. Moreover, the inclusion of Si improves the adhesion and enhances the thermal stability of DLC layers, and the addition of the elements F, Ag, or Si leads to improvements in hemocompatibility. The results showed that adding silicon to DLC enhanced its hemocompatibility and enhanced the seeding of endothelial cells onto Si-doped DLC surfaces. Moreover, the effectiveness of seeding endothelial cells was increased by thermally annealing Si-DLC films. Dopants such as Ti and Cr increase corrosion resistance while also decreasing internal stress [4,16,17,18,19,20]. When compared with a pure DLC coating, the low addition of copper (1.2%) or titanium (2.75%) dramatically increases wear resistance. Moreover, it has been found that titanium has a stronger effect than copper. For coatings doped with nitrogen, compared with pure carbon coatings, lower coefficients of friction and about 3–10 times longer lifespans were found. Furthermore, it has also been noted that nitrogen doping decreases residual stress and increases the thermal stability of carbon films [21]. Research indicates that the addition of Si can escalate the sp3 fraction and the matrix’s binding force. Including nitrogen elements can facilitate the creation of the sp2 ring, and silicon and nitrogen combine to form a stable microstructure [22]. Aluminum doping impacts corrosion behavior. Furthermore, the corrosion potentials of diamond-like carbon coatings tended to decrease toward more negative values with increased aluminum content. Li et al. [23] investigated the structural and mechanical properties of vanadium and nitrogen co-doped diamond-like carbon (DLC–VN) films. Their results indicated that the tested films possessed higher hardness in contrast to pure DLC films [23,24].

It is possible to coat DLC on polymer surfaces to obtain highly functionalized materials. DLC films deposited on such polymer substrates have been studied and used for applications as gas barrier materials and biocompatible medical devices [12]. The aim of this review is to summarize the current knowledge and research regarding diamond-like carbon coatings.

## 2. Deposition Techniques for DLC Film

It is common knowledge that deposition techniques impact DLC characteristics [9,25]. DLC is deposited using vacuum environment reaction techniques, such as physical vapor deposition and chemical vapor deposition. For DLC film deposition by PVD techniques, solid carbon is evaporated and the carbon vapor produced deposits on a substrate. CVD is a chemical process that is the opposite of PVD. CVD is used to deposit solids from gases, in which a source gas is decomposed by plasma irradiation, heating, or photoexcitation and a thin film is deposited. In DLC film deposition by CVD methods, hydrocarbon gas is decomposed and the carbon vapor produced deposits on a substrate. The CVD method using plasma for hydrocarbon deposition is plasma-assisted chemical vapor deposition (PACVD). This method is useful because it can be used to deposit DLC films on both conducting and insulating substrates at room temperature [7,26,27]. PACVD permits the covering of objects with complex geometries, offers superior control over the film stoichiometry, and minimizes variations in film thickness as compared with the PVD technique. These features can improve the quality and life cycle of the components used in biomedical applications. Furthermore, the coating’s uniformity in terms of thickness and chemical composition directly affects how DLC films respond to bodily fluids [1,28].

Other variations of the PACVD technique are the radiofrequency PACVD, bipolar PACVD, glow-discharged PACVD, and pulsed direct current (DC) PACVD. The pulsed DC PACVD is the simplest, most affordable, and novel method, making it simple to implement on an industrial scale. CVD also includes microwave plasma chemical vapor deposition (MPCVD) and plasma-based ion implantation. The advantages of MPCVD include the lack of pollution, good film-forming quality, and easy operation [1,25,29,30,31].

It is also possible to use a modification of the known CVD method for DLC deposition. Hot filament chemical vapor deposition (HFCVD) is based on the dissociation of precursor gases near a hot fiber and the formation of a film on a substrate kept at a much lower temperature than that in the classical chemical vapor deposition method. The relatively low temperature allows the processing of thermally sensitive materials [32,33,34,35,36].

Ion beam deposition, pulsed laser deposition, filtered cathodic vacuum arc (FCVA), high-power impulse magnetron sputtering (HiPIMS), and magnetron sputtering and laser ablation are all examples of physical vapor deposition [1,37,38]. However, due to the low homogeneity in the thickness and low deposition rate, some PVD techniques have significant disadvantages when depositing DLC films for biomedical applications. This may cause metal ions to be released from the substrate, which may have potentially hazardous long-term consequences. Additionally, the application of targets may result in significant clusters on DLC films, which restricts the possibility of homogeneous doping with nanoparticles and achieving appropriate properties supplied by dopants [1].

Hydrogen-free coatings can be produced by using magnetron sputtering with ion plating, FCVA, excitation in the high-frequency field (RFCVD—Radio Frequency Chemical Vapor Deposition), and pulsed cathodic arc evaporation. They exhibit better thermal stability compared with hydrogen-containing coatings, while the method of pulsed cathodic arc evaporation ensures obtaining coatings at a higher speed, reducing their roughness, and reducing tribological wear due to the reduction in the share and dimensions of build-up in the form of atomized drops characteristic of the arc method [14].

Scientists are still working on new methods under the conditions of deposition of diamond-like carbon coatings. Wen Zhu et al. [29] deposited DLC films on 316 L stainless steel. In order to improve biocompatibility, they changed parameters such as the temperature, CH4:H2 flow rate, and deposition time in a microwave plasma chemical vapor deposition system. As a result, it was found that the structure of the coating progressively became more compact and smooth, and the structure of the graphite phase of the film tended to change to the diamond phase when the deposition temperature increased and the gas flow rate decreased. The best parameters of the film-forming process were as follows: temperature of 900 °C, gas flow ratio of CH4:H2 of 10:190, and deposition time of 60 min. Consequently, coated 316 L stainless steel had better osteogenesis ability and biocompatibility compared with uncoated 316 L stainless steel [29].

## 3. Surface Preparation

For better adhesion of the layer to the substrate, appropriate methods of surface preparation should be used, changing the shape of the crystallographic lattice of the surface material to one closer to the crystallographic lattice of diamond by nucleation. The layer deposited on a diamond-containing substrate may be homoepitaxial or even have a monocrystalline diamond structure as opposed to a heteroepitaxial or polycrystalline layer. In the case of materials that do not contain diamond in their structure, there is a need for additional surface preparation, because diamond does not grow spontaneously on such substrates. Moreover, even for silicon substrates that have the closest crystal lattice, the growth process without nucleation is very heterogeneous. The nucleation process affects the thickness of the layer, the size of the crystallites, the homogeneity, morphology, surface roughness, defects, and adhesion of the layer to the substrate [39].

Heterogeneous nucleation methods include mechanical and dry nucleation, i.e., scratching with dry diamond powder. Electric nucleation is nucleation in an electric field. Wet mechanical nucleation involves the use of a suspension with nanodiamond particles, an ultrasonic bath, centrifugation, and immersion [39,40]. Interlayer deposition is a frequently used method to improve the adhesion of the diamond layer. Utilizing an interlayer serves several purposes, such as improving the nucleation of diamond crystals, enhancing adhesion, reducing thermal residual stresses, and providing a compatible structure for the nucleation of diamond crystals [40].

Currently, the most efficient and widespread method of nucleation is mechanical nucleation by an ultrasonic bath in suspensions containing diamond particles in water or dimethyl sulfoxide (DMSO). The typical grain size of the nanodiamond powder used for suspension is 4–5 nm. Diamond suspensions based on DMSO with the addition of polyvinyl alcohol to increase the viscosity of the substance enables the use of centrifugation and immersion methods, which allow for the quick and efficient nucleation of various substrates, i.e., silicon, quartz, and titanium [39].

However, because they all exhibit extremely low critical load values, there is a problem with the adhesion of DLC films to steels and metal alloys. The main issues with its application include internal stresses, inadequate toughness, inadequate adhesion, and a high film-to-substrate-stiffness ratio, which lead to the failure of films [24,41].

Another important way to increase adhesion involves the use of vapor deposition and inserting an interlayer between the substrate and the diamond coating. The interlayer mediates the thermal expansion divergency between the substrate and the coating, in addition to preventing transition metal diffusion. Monolithic layers or multilayers as well as pure metals, nitrides, carbides, silicates, oxides, or borides can be used as interlayers [42]. Several interlayers have been studied and scientists have shown that layers such as CrN, TiN, SiC, Cr/CrC, TiC, and TiCN increase film adhesion and surface coating. Furthermore, duplex diffusion and the functionally graded multilayer design are some innovative approaches that may enhance coating durability [43,44].

According to numerous authors, the Ti6Al4V alloy requires the use of a silicon interlayer in order to produce diamond-like carbon films with acceptable adhesion. Moreover, DLC coatings are unfortunately easy to peel off when deposited on the Ti6Al4V substrate due to the mismatch between the mechanical and thermal properties of diamond-like carbon coatings and Ti6Al4V materials, which has limited the application of DLC coatings. Numerous methods have been tested to solve the aforementioned problems, including the optimization of coatings’ characteristics, the addition of components, the addition of intermediate layers, and substrate pretreatment. In terms of competitive advantages for coating preparation, surface texturing has the capacity to create geometric textures with various morphologies, increase the surface contact area, provide a good adhesion interface, and afford mechanical locking ability to the coatings [45]. It has been reported that adding a specific texture to a surface, such as dimples or pillars, might significantly alter its hydrophobicity and tribological performance [46,47].

An advanced surface engineering technique that can improve tribo-performance is laser surface texturing (LST), which is used, for instance, in the articulation of hip implants [48]. Laser texturing prior to DLC deposition can effectively increase the adhesion between the coating and the substrate. The strength of this bond can also be increased by the implantation of carbon ions. The synergistic combination of texturing and carburizing can effectively improve the tribological performance of the coatings and also increase the wettability of the material [45,49].

**Figure 1:**
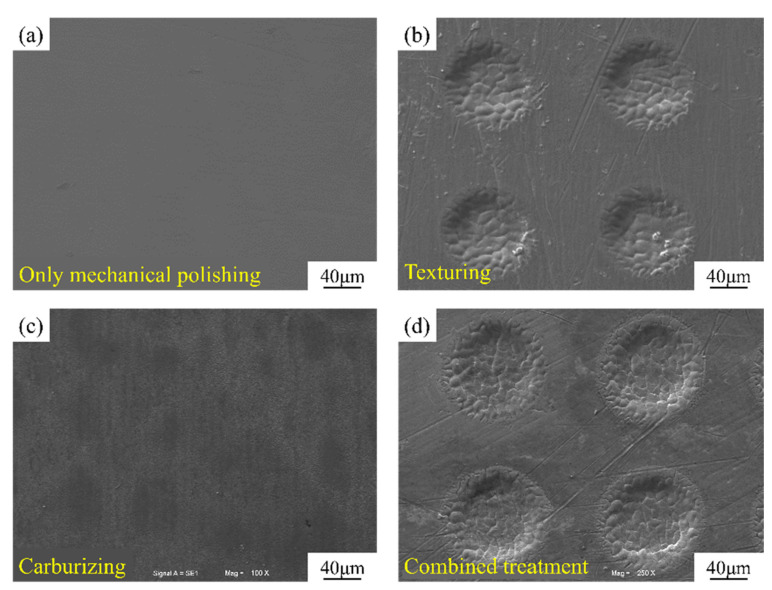
Different surface treatments shown in SEM images: (**a**) only mechanical polishing, (**b**) texturing, (**c**) carburizing, and (**d**) combined treatment of texturing and carburizing (reprinted from [45], permission from Elsevier, license number: 5510710732134).

**Figure 2:**
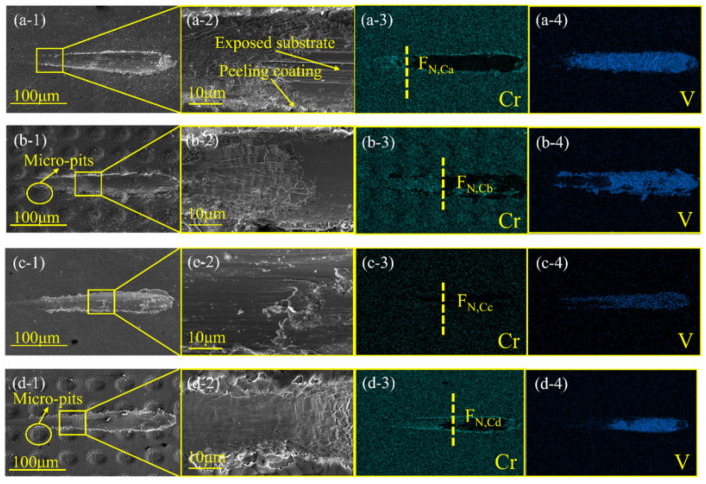
SEM and EDS analyses of different surface treatments after scratch tests: (**a-1**–**a-4**) only mechanical polishing, (**b-1**–**b-4**) texturing, (**c-1**–**c-4**) carburizing, and (**d-1**–**d-4**) combined treatment of texturing and carburizing (reprinted from [45], permission from Elsevier, license number: 5510710732134).

Dong B. et al. [45] studied the effects of texturing, carburizing, and their combination on enhancing the bonding strength of the DLC film on a Ti6Al4V alloy substrate. Both the specific surface of the substrate and the wettability could be increased by coupling the mechanical–physical–chemical effects accompanying laser texturing and the carburizing reaction. This ensured mechanical self-locking and matching chemical properties between the Ti6Al4V substrate and DLC coatings. Figure 1 shows micrographs of the substrate’s surface with different surface treatments. Moreover, Figure 2 displays scanning electron microscopy (SEM) micrographs and energy-dispersive X-ray spectroscopy (EDS) analyses of various surface treatments after a scratch test. The bonding strengths of hybrid pretreated coatings were the highest compared with the other three samples [45].

Additionally, amorphous carbon films have a wide range of wettability options. Amorphous carbon films that have been modified by doping with fluorine or aluminum can have contact angles of 105°. Super-hard amorphous carbon films can be deposited using the laser arc method. These films have also broad structural variability. Within clearly defined matter, the wettability can be changed. The wettability can be modified in a specific way by altering the deposition conditions with the use of doped carbon targets and unique residual gas atmospheres. This method allows for the deposition of amorphous carbon films with contact angles ranging from 40° to 120° [50,51].

It is commonly believed that the lower the coating thickness, the higher the load-bearing capability, but the critical load is significantly influenced by various failure mechanisms. The higher the coating thickness, the better the coating durability, provided that the internal stress of the coating is lower than the stress developed by the applied load. This is due to the fact that higher coating thickness provides better stress shielding for softer substrates by increasing the critical load limit that initiates coating failure and protects the substrate from plastic deformation [44,52].

Furthermore, the incorporation of metal in diamond-like carbon film increases the disorder. The bonding of doping elements with carbon results in the formation of Ti, Al, and V carbides on DLC. The internal stresses and adhesion can be improved by metal doping, and, as a result, the tribological properties of metal-doped films are superior to those of metal-free films [24].

## 4. Biomedical Application for DLC Film

DLC films have been studied over the last two decades, especially for their antibacterial, tribological, and corrosion properties [41]. The advantages of diamond-like carbon coatings include their outstanding mechanical qualities, low coefficient of friction, anti-wear properties, strong adhesion to the substrate, and biocompatibility. In simulated body fluid, amorphous carbon has exhibited bio-inertness and a non-toxic response toward osteoblast-like cells [53,54,55,56,57]. Furthermore, DLC films can improve endothelization on medical implants and reduce thrombotic clots [29]. Due to these characteristics, it is perfect as a coating for orthopedic, dental, and cardiovascular applications. DLC coatings are applied to a variety of items, including dental implants, intraocular lenses, stents, catheters, and heart valves [13,53,54,58,59]. To expand their applications, the protective coatings’ hydrophobicity and wear resistance should be further enhanced. Such coatings can be widely used in non-stick surgical tools, such as electrosurgical knives and radio-frequency scalpels if the hydrophobicity is increased. The reliability and durability of human implants can also be improved if the friction and wear behavior are further enhanced. Traditionally, doping with other elements has been used to enhance the properties of DLC coatings. For instance, increasing the hydrophobicity of DLC coatings has been most accomplished by adding F [46]. Nakamura T. [60] compared fluorine-modified carbon materials to their unmodified counterparts. The fluorine-modified carbon materials showed a lower friction coefficient and a decrease in their surface energy, evaluated by the contact angle using water. In contrast to the untreated diamond film, which had a contact angle of 81°, the modified diamond film had a contact angle of 118°. The fluorine-modified diamond surface was discovered to have strong water-repellent properties [60].

**Figure 3:**
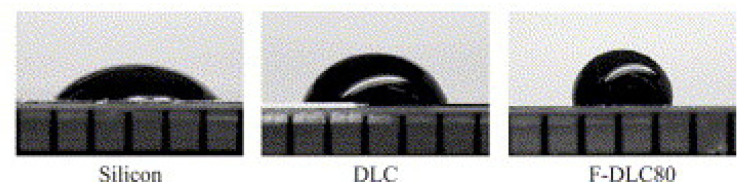
Contact angle measurements of 10 µL human whole-blood droplets on the sample surface. F-DLC80 showed the highest hydrophobicity (reprinted from [61], permission from Elsevier, license number 5510710188982).

**Figure 4:**
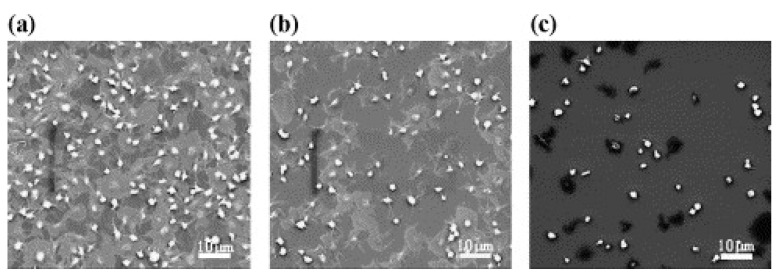
Morphology of adherent platelets on (**a**) Si, (**b**) diamond-like carbon, and (**c**) F-doped diamond-like carbon surfaces (60 min incubation in platelet-rich plasma) observed using SEM (reprinted from [61], permission from Elsevier, license number 5510710188982).

There have been numerous reports indicating a connection between surface energy, wettability, and cell adhesion on DLC films. They reported that hydrophobic surfaces frequently inhibited the adsorption of blood cells. Regarding hydrophobicity, fluorocarbon polymers are well known for having excellent water-shedding characteristics [61,62,63]. Saito T. et al. [61] investigated the influence of doping with fluorine on diamond-like carbon films on their anticoagulant properties by changing their content. F-DLC films were prepared on silicon substrates using RFCVD by changing the ratio of hexafluoroethane and acetylene. On Si, DLC, and F-DLC, human whole-blood-droplet contact angles of 24.2°, 60.8°, and 95.3° were measured, respectively. Droplet images are shown in Figure 3. Moreover, the platelet adhesion and activation on the surface of F-DLC films incubated with platelet-rich plasma were dramatically reduced. SEM and statistical analyses showed that the antithrombogenicity of DLC coatings significantly increased with the addition of fluorine (Figure 4) [61].

Additionally, in order to allow simple tooth movement in orthodontics, it is critical to reduce static friction between brackets and wires. Akaike S. et al. [64] deposited diamond-like carbon films, fluorine-doped DLC (F-DLC), and silicon-doped DLC (Si-DLC) coatings onto the slot surfaces of stainless steel orthodontic brackets using the PACVD method and characterized the frictional properties between the coated bracket and wire under dry and wet conditions. In every specimen, the contact angle was smaller against PBS or distilled water than it was against distilled water. In comparison with DLC, Si-DLC was more hydrophilic, while F-DLC was significantly more hydrophobic [64]. The reduction in the value of the contact angle in the case of silicon doping was due to the increase in surface energy resulting from the electron-donative properties of C–Si. However, it is believed that C–F and C–CF bond polarization on the topmost surface of F-doped DLC will decrease the surface energy and increase the contact angles [64,65,66]. Every DLC coating was substantially harder than stainless steel. The surface hardness of the DLC decreased when fluorine or silicon was included in it. In comparison with DLC, fluorine or silicon doping both considerably decreased Young’s modulus. According to the results of their research, DLC, F-DLC, and Si-DLC coatings significantly reduced static friction [64].

One of the most popular orthopedic procedures is arthroplasty. However, the revision rates for patients can be as high as 10% [61]. The main reasons for implant failure are mechanical damage, cellular damage, infections, and blood coagulation (which could result in thrombosis). The interaction of implants with the body’s cells is very important, because implants should prevent uncontrolled cell growth, preserve their integrity inside the body, and avoid debris formation in order to perform their role. For improved bone–implant integration in the case of joint implants, osteoblast (bone-forming cells) attachment is preferred. Additionally, the implants should not cause infections [67,68,69,70,71]. The parameters of a good biomaterial for orthopedic implants fit well with good wear behavior, corrosion resistance, chemical inertness, and excellent smoothness [72]. Therefore, DLC films are particularly interesting options for joint prostheses because of their chemical inertness, acceptable mechanical qualities, biocompatibility, and outstanding tribological properties. DLC films can be doped with substances, including chromium, titanium, copper, and silver, to overcome this problem. Ag doping specifically improves the hardness and adhesion of DLC films, as well as their wear resistance and antibacterial characteristics [63,64,65,66,67,68,69,70,71,72,73,74,75,76].

Whole hip or knee arthroplasties may benefit from having diamond-like carbon coatings applied to the articulating surfaces to improve their biotribological behavior and longevity. Rothammer B. et al. [77] investigated the effects of the choice of the coated component (metallic or polymeric), as well as the variations between a higher and lower load case with, respectively, non-cross-linked and typically cross-linked ultrahigh-molecular-weight polyethylene. Excellent mechanical characteristics with a notable improvement in the indentation hardness and elastic modulus ratios were present in the examined coating systems. The coatings’ potential for use in total joint arthroplasties was confirmed by the fact that their adherence to polymeric and metallic substrates, as assessed by modified scratch tests, could be rated as very good and as satisfactory, respectively. Owing to the significantly higher roughness, the coatings mostly increased friction, although wear was greatly decreased [77]. Kopova I. et al. [72] used a titanium gradient interlayer (Ti/Ti-C:H) to evaluate the wear of a trapeziometacarpal total joint arthroplasty implant, which was composed of DLC-coated Co-Cr-Mo alloy, and to assess the potential cytotoxicity of the wear particles produced by simulated loading. After 3 million cycles of increasing the loading force up to 2.5 kN, there was no discernible wear or delamination of the DLC coating. Moreover, human osteoblast-like cells were used to test the cytotoxicity of DLC wear debris in vitro. Thus, they concluded that the simulated loading of the trapeziometacarpal total joint replacement implant did not result in the formation of any cytotoxic wear debris [72].

Research on the impact of DLC coatings on macrophages, fibroblasts, and osteoblast-like cells, which are found in the tissues surrounding a total joint replacement, did not discover any indication that the coatings could cause cytotoxicity [70,78]. The biocompatibility of DLC with blood monocytes has also shown promising results, which is significant because monocytes regulate inflammatory processes and might affect the osseointegration of implants [70,79]. Moreover, research on coatings in animal organisms has also been performed. Hajduga et al. [80] compared the influence of titanium implants with TiN, ZrN, and DLC a-C:H coatings on animal organisms. Their research did not show any significant differences between the examined materials. There were no foci of necrosis or inflammatory responses brought on by the implant in any of the three cases. All of the coatings that were examined showed good biocompatibility, but had different tribological properties [80].

Katouno J. et al. [81] manufactured a DLC that contained Zn (Zn-DLC) using the reactive sputtering method. Zinc has attracted attention because it increases alkaline phosphatase (ALP) production, which promotes bone calcification that can enhance osteogenesis. Osteoblasts cultured on Zn-doped DLC tended to show a greater area of calcification than those cultured on diamond-like carbon, although no important differences in alkaline phosphatase activity were noticed [81]. Saito K. et al. [82] examined whether Zn-DLC could be used for treating bone fractures in vivo. Using computed tomography-created 3D models of the fractured parts, they compared the healing stages of the bone fractures between the Zn-doped DLC splints group and the diamond-like carbon splints group. The numbers of mice that were completely treated by 4 weeks when fixed with DLC and Zn-doped DLC splints were two and four, respectively. Following the in vivo test, they examined the bone features, such as bone volume, bending and twisting strength, cortical bone thickness, cortical bone area ratio, and spongiosa bone density. These findings suggested that Zn-doped DLC could supply Zn under in vivo conditions and may enhance the therapeutic effect of traditional implants used in the treatment of fractures [82].

The primary global cause of morbidity and mortality is atherosclerosis. It is a vascular intima disease that can affect any part of the circulatory system, including the aorta and coronary arteries, and is characterized by intimal plaques. An important part of the etiology of atherosclerosis is inflammation [83,84,85,86]. Before the arteries are expanded, stents are placed in the clogged arteries to help with their opening and the restoration of blood flow [12]. After percutaneous coronary intervention (PCI), patients must take the recommended dosage of an antiplatelet medicine prescribed by the physician [87]. The majority of acute PCI problems are caused by platelet activation, making medicines that prevent platelet aggregation essential to the procedure’s safety. Early complications include hemorrhage from the arterial access site (reduced by a radial approach). Abrupt vessel closure, stroke, vessel perforation, and tamponade are uncommon [88]. The treatment options for atherosclerosis may include angioplasty and artery stenting. Although the metals from which stents are made have great mechanical properties, they still have some disadvantages, i.e., restenosis, stent-associated thrombosis, and occlusion. The main side effects of stents are related to the release of metal ions, e.g., thrombosis and allergic reactions, which should be prevented [12]. The usual competent endothelium structure is unavoidably disturbed by the deployment of a drug-eluting stent (DES). This is further worsened by the fact that non-selective cytostatic or cytotoxic drug elution significantly lowers the quality of endothelium regeneration and vessel repair. Chronic inflammation, platelet adhesion, and hypersensitivity reactions occur when the metal struts of stents are exposed to the bloodstream. In addition, the stented segment has accelerated neoatherosclerosis because of improperly formed endothelial cell connections and decreased barrier function, which allow lipoproteins to enter the sub-endothelial space [89,90]. It is, therefore, effective to cover the stent’s surface with biocompatible and protective materials, DLC has received a lot of attention as such a coating material, generating a number of promising outcomes [12,91].

Platelets, especially active platelets, are connected to the thrombogenicity of stents. Blood chemistry and the nature of the material’s surface control the mechanism of blood coagulation. Implants or other medical devices that are in contact with blood should be hemocompatible. Hemocompatible materials should perform minimal hemolytic activity. This means that the material does not cause any damage or changes to the configuration, morphology, and stability of morphotic blood components [12,58,92,93]. The advancement of biocompatibility, combined with technological innovations in interventional procedures, such as intravascular ultrasound-guided stenting and high-pressure insertion techniques, has been the primary goal of efforts to further reduce stent-associated thrombosis and in-stent restenosis. The thrombogenicity of stent metal is one issue with vascular stenting. All of the stents that are on the market right now are made of metal, e.g., stainless steel or nickel–titanium alloy (nitinol). The surface of an uncoated stent can release metal ions, including nickel and chromium, which may have enhanced platelet and leukocyte activation in the surrounding tissue [12]. Numerous examples demonstrate the good biocompatibility of DLC and the reduced platelet adhesion and activation linked to the high albumin/fibrinogen adsorption ratio on DLC surfaces. These investigations have shown that DLC does not cause any harm to specific living cells, loss of cell integrity, or any inflammatory reaction. Moreover, DLC decreases platelet adhesion and activation, according to an in vitro study [12,94].

The anti-thrombogenicity of biomaterials for medical applications can be improved by studying polishing techniques for material surfaces. Many stent manufacturing companies consider electrochemical polishing to be essential for their products [8,95,96]. Hasebe T. et al. [8] evaluated the effects of the surface roughness of coatings on thrombus formation. On three different roughness-controlled polycarbonate substrates, DLC and F-DLC films were applied, and platelet adhesion and activation were studied on each substrate. The surface roughness of DLC-coated polycarbonate (PC) and F-doped DLC-coated PC ranged from 4.1 nm to 97 nm. There were no notable variations in the platelet-covered area within this range. However, the evaluation of the F-DLC films demonstrated significant reductions in platelet adhesion and activation when compared with diamond-like carbon coatings for every grade of roughness. This suggests that the chemical properties of the surface, such as wettability, interfacial free energy, and higher ratios of albumin/fibrinogen adsorption, might be more significant in the mechanism of F-doped DLC non-thrombogenicity [8]. In the scientific literature, one can find several methods for surface modification. For example, a carbon film was deposited on Co–Cr alloy stents by physical vapor deposition at low temperatures (T < 100 °C), resulting in a very high adhesive strength to the material. Biological tests were performed at 7, 30, and 180 days to observe the inflammatory responses and confirm rapid endothelization. Modern DLC-coated Co–Cr stents showed more thorough and uniform endothelization, as well as effective prevention of fibrin deposition and platelet activation, which lowered the risk of thrombotic clots. Within 30 days, vascular healing was completely stabilized as a result of the coating’s reduced inflammatory activation and, at 180 days, there was only a limited amount of neointimal proliferation [53].

About 25–30% of today’s heart issues are caused by heart valve dysfunction. Artificial heart valves must be implanted to replace faulty ones through sophisticated and sometimes risky surgery. However, once a heart valve has been replaced with an artificial one, it is desirable that it lasts for the life span of the patient. Any method that can improve the operational life of artificial heart valves is, therefore, greatly desired. Ali N. et al. [97] studied chromium-modified diamond-like carbon for potential applications in mechanical heart valves. Using a magnetron sputtering technology and an intensified plasma-assisted processing system, Cr-DLC samples were created. Studies of the biological response of human microvascular endothelial cells to the evaluated material showed that changes in the Cr content in Cr-DLC films affect the adhesion and growth of endothelial cells [20].

Kwok et al. [97] investigated the blood compatibility of C–N. N-doped diamond-like carbon films were received by plasma immersion ion implantation–deposition by operating a carbon-filtered cathodic arc source in concert with N/Ar plasma. Their findings indicated that low-temperature isotropic pyrolytic carbon was less biocompatible than N-doped, hydrogen-free amorphous carbon coatings with the proper N concentration. Unfortunately, a film’s blood compatibility is actually reduced by an unusually high nitrogen content [97].

## 5. Antibacterial Properties of Doped DLC

A significant clinical problem today is the development of bacterial biofilms on the surface of medical equipment. Numerous illnesses are brought on by bacteria’s highly adaptable ability to colonize the surface of biomaterials [67]. Modified DLC films have been shown to have better antibacterial characteristics, biocompatibility, stability, and cell adhesion in recent experiments using silver and titanium dioxide [3,41].

It has long been known that silver provides strong antibacterial properties against a wide range of microorganisms. In addition, silver has the ability to inhibit polybacterial colonization. The primary bactericidal activity of Ag is carried out through the release of Ag^+^ ions via an oxidative reaction in an aqueous solution or biological medium [3,98,99]. Ag-doped DLC coatings’ stability and long-term antibacterial activity will also depend on many aspects, including surface energy, roughness, the coating’s micro- and nanostructure, and the concentration and distribution of silver throughout the coating’s thickness (dispersed or agglomerated in the form of nanoparticles). Doping DLC films is challenging [3,41]. Many techniques, including pulsed laser deposition, ion beam deposition, plasma-accelerated CVD, and FCVA, have been used to create Ag-DLC films [37,100,101,102,103].

Bociaga D. et al. [67] studied a special nanocomposite Ag-doped (by ion implantation) diamond-like carbon coating prepared by hybrid radio frequency/magnetron sputtering plasma-assisted CVD. A uniform distribution of Ag ions in the amorphous diamond-like carbon matrix, good biocompatibility when in contact with mammalian cells, and elevated levels of bactericidal properties were all evident in the examined coatings [67].

Onodera S. et al. [104] examined the correlation between the physical and antibacterial properties of a F-containing DLC. Fluoride is known for its ability to inhibit bacterial glucose metabolism. A F-doped DLC film was created using a polystyrene substrate by plasma-assisted CVD with CH_4_ and C_2_F_6_ as source gases. Then, after the formation of the fluorine-containing DLC layer, plasma treatment at atmospheric pressure with gaseous oxygen was performed to improve its biocompatibility. In addition to the antibacterial properties of oxygen and the inhibitory effect of fluorine on the energy metabolism of glucose by bacteria, the physical changes produced, such as the smoothing of the outermost surface and the hydrophilic contact angle, increased the antibacterial properties of inhibiting bacterial adhesion and increasing the contact surface with bacteria [104].

Harrasser N. et al. [105] assessed the antibacterial effectiveness of various surface treatments on a Ti alloy, which had been conversed to diamond-like carbon (DLC-Ti) and doped with high and low concentrations of Ag with a modified ion implantation technique. Clinically relevant bacterial strains (Staphylococcus epidermidis, Staphylococcus aureus, and Pseudomonas aeruginosa) were grown planktonically on Ag-DLC-Ti and their adhesion was compared with that to untreated Ti. The research results confirmed the antibacterial properties of silver-doped coatings. Increasing the concentration of silver improved the antibacterial properties. Additionally, no influence of Ag-DLC-Ti on osseointegration was observed. There is evidence that coating Ti with DLC can improve osseointegration [105].

Another approach is the use of nanodiamonds (NDs). Owing to their unique properties, including hydrophilic qualities, high surface area, hardness, high thermal conductivity, corrosion resistance, low toxicity, high chemical stability, antibacterial properties, and the presence of various functional groups on the surface (which makes the functionalization process much more convenient), ND has attracted much attention [106,107,108,109].

To create new materials and new applications, ND can be used as a dopant material in DLC films. The process of detonating heavy explosives under high pressure and temperature mostly produces ND [41,110]. Detonation nanodiamond (DND) is a material that has been applied as a nanoantibiotic by numerous medical researchers. Gutiérrez J.M. et al. [41] examined DLC films doped with nanodiamond; coatings were deposited on Ti6Al4V substrates. Gram-negative Escherichia coli was used in an antibacterial test. The antibacterial activity was tested and, 6 h after direct contact with the film, the antibacterial activity was close to 95%. This finding indicates that DLC is a superior material for biomedical applications. Nearly 25% of antibacterial activity was seen after 18 h of direct contact with the doped DLC film, suggesting that nanodiamonds are able to preserve their antibacterial properties and damage E. coli cells [41].

## 6. Conclusions

DLC coatings are commonly used in medicine, both in medical implants and for the production of medical instruments. The surfaces of artificial heart elements in contact with blood are covered with DLC coatings owing to the exceptional hemocompatibility of the coating. The unique properties of the coating depend on the sp^2^/sp^3^ phase ratio. Owing to its sp^2^ and sp^3^ bonding, DLC, an amorphous form of carbon, possesses both diamond and graphite qualities. Additionally, as a result of its structure, it is extremely hard, chemically relatively inert, and has an extremely low friction coefficient. Additionally, DLC coatings can stimulate the rapid endothelization of implants with impaired platelet activation, reducing thrombotic clots.

Techniques that use vacuum environment reactions to deposit DLC include physical vapor deposition and chemical vapor deposition. However, it is also possible to use modified methods, e.g., radio frequency plasma-assisted chemical vapor deposition or hot-filament CVD. In order to improve the adhesion of the layer to the substrate, appropriate methods of surface preparation should be used, changing the shape of the crystallographic lattice of the surface material to one closer to the crystallographic lattice of diamond by nucleation.

To obtain additional properties, DLC films can be enriched with chemical elements, including Ag, F, P, Si, and Ti. Among the dopants used in biomedical applications, silver is the most commonly used element due to its antimicrobial properties. Increasing the concentration of silver improves antimicrobial properties. In addition, antibacterial properties were achieved by admixing DLC with fluorine, which inhibited the adhesion of bacteria. Antimicrobial activity was also found in nanodiamonds, which further improved film adhesion. In addition, metal doping improved adhesion and reduced internal stresses, resulting in metal-doped films exhibiting better tribological properties than metal-free films.

Diamond-like carbon coatings, according to the current literature, are the most promising coatings for biomedical applications, but other applications, such as automotive and adhesives, are increasingly expected.

## Data Availability

No new data were created or analyzed in this study. Data sharing is not applicable to this article.
